# Phosphorylation-Mediated Regulation of Alternative Splicing in Cancer

**DOI:** 10.1155/2013/151839

**Published:** 2013-08-28

**Authors:** Chiara Naro, Claudio Sette

**Affiliations:** ^1^Department of Biomedicine and Prevention, University of Rome “Tor Vergata”, 00133 Rome, Italy; ^2^Laboratories of Neuroembryology and of Cellular and Molecular Neurobiology, Fondazione Santa Lucia IRCCS, 00143 Rome, Italy

## Abstract

Alternative splicing (AS) is one of the key processes involved in the regulation of gene expression in eukaryotic cells. AS catalyzes the removal of intronic sequences and the joining of selected exons, thus ensuring the correct processing of the primary transcript into the mature mRNA. The combinatorial nature of AS allows a great expansion of the genome coding potential, as multiple splice-variants encoding for different proteins may arise from a single gene. Splicing is mediated by a large macromolecular complex, the spliceosome, whose activity needs a fine regulation exerted by *cis*-acting RNA sequence elements and *trans*-acting RNA binding proteins (RBP). The activity of both core spliceosomal components and accessory splicing factors is modulated by their reversible phosphorylation. The kinases and phosphatases involved in these posttranslational modifications significantly contribute to AS regulation and to its integration in the complex regulative network that controls gene expression in eukaryotic cells. Herein, we will review the major canonical and noncanonical splicing factor kinases and phosphatases, focusing on those whose activity has been implicated in the aberrant splicing events that characterize neoplastic transformation.

## 1. Introduction

In eukaryotic cells, the expression of each gene is finely tuned by a complex network of regulative processes affecting all steps of transcript maturation, from nuclear transcription to cytosolic export and utilization of the mRNA. A crucial step in this regulative network is represented by pre-mRNA splicing, the molecular process that mediates the removal of intronic sequences and the joining of exons. What makes splicing an outstanding player in controlling gene expression is its flexibility, which allows a remarkable increase of the coding potential of the genome through alternative selection of exons. Indeed, alternative splicing (AS) allows each gene to encode for several coding and noncoding mRNA variants, which often display different activities and/or patterns of expression. AS is, therefore, one of the principal mechanisms underlying the well-known discrepancy between increasing organismal complexity and content of genes contained in the genome [1]. In line with its central contribution to genome complexity, it is estimated that up to 90% of human multiexon genes undergo AS [2], and the importance of this regulative mechanism for both developmentally regulated and pathological cellular processes is now well recognized (reviewed in [3]).

The splicing process is carried out by the spliceosome, a complex macromolecular machinery composed of five small nuclear ribonucleoprotein particles (U1, U2, U4, U5, and U6 snRNPs) and more than 200 auxiliary proteins. The spliceosome mediates the recognition of the short consensus sequences surrounding the 5′-(GU) and the 3′-(AG) splice site and catalyzes the two transesterification reactions necessary for the removal of the intron and ligation of the selected exons (reviewed in [4]). Due to the degenerate nature of the sequence elements recognized by the spliceosome, its recruitment to the maturing pre-mRNA requires the action of both *cis-*acting RNA sequence elements and *trans*-acting RNA binding proteins (RBPs), such as Ser/Arg (SR) rich proteins, heterogeneous nuclear ribonucleoproteins (hnRNPs), and splicing factors belonging to other RBP families. In addition, AS is also regulated by mechanisms acting both co- and posttranscriptionally, through epigenetic modifications of the chromatin, regulation of the RNA polymerase II (RNAPII) transcription rate, and posttranslational modifications of both spliceosome components and auxiliary splicing factors, among which reversible phosphorylation acts as a major player. 

## 2. Impact of Phosphorylation on the Catalysis of Splicing

A proper regulation of the phosphorylation status of the spliceosomal proteins and of accessory splicing factors is crucial for the correct regulation of both constitutive and AS events. Early studies already described the importance of a correct balance between phosphorylation and dephosphorylation events in the splicing process by showing that both activation [5] and inhibition [6] of the PP1 and PP2A phosphatases are required for splicing catalysis. Several reports have then further highlighted the importance of regulated phosphorylation events for the correct assembly and catalytic activation of spliceosomal components, such as PRP28 [7], PRP6, or PRP31 [8]. Equally, dephosphorylation events, such as those regarding the U5 and U2 snRNP component, U5-156 kDa and SAP155 [9, 10], were shown to be essential for spliceosome activity, proving the importance of subsequent rounds of phosphorylation and dephosphorylation events in the regulation of the splicing process.

Regulative phosphorylation and dephosphorylation events concern not only the spliceosomal components but also some accessory RBPs that cooperate with the spliceosome in the selection of splice sites. For example, phosphorylation of the splicing factors SF1 and SRSF1 (prototypic SR protein previously known as ASF/SF2) modulates their interaction with U2AF65 and U1snRNP, respectively, thus modulating spliceosome assembly [11, 12]. The dynamic phosphorylation/dephosphorylation of SR proteins is particularly relevant for the regulation of their functions, as both hypo- and hyperphosphorylation can inhibit splicing [13]. For instance, phosphorylation of SRSF1 promotes spliceosome assembly, whereas its dephosphorylation is necessary for the catalysis of the first transesterification reaction [14]. 

## 3. Phosphorylation and Splicing Factors

SR proteins are a family of nuclear RBPs involved in the regulation of both constitutive and AS, whose activity is greatly modulated by reversible phosphorylation. Their structure is generally characterized by two N-terminal RNA recognition motif (RRM) a C-terminal region enriched in Arg-Ser residues (RS domain), which are the main targets of regulative phosphorylation. Phosphorylation of the RS domain of SR proteins has a great impact on their functionality, as it may affect their binding to target mRNAs, their interaction with other proteins and their intracellular localization. As an example, binding of SRSF5 (previously reported as SRp40) to its high-affinity RNA-binding site is strictly dependent on the phosphorylation of its RS domain [15]. One of the most significant examples of how phosphorylation may affect the splicing activity of SR proteins is represented by SRSF10 (previously known as SRp38). This SR protein acts as a specific splicing activator in its RS-phosphorylated form [16], whereas dephosphorylation converts it into a potent splicing repressor [17]. Notably, dephosphorylation of SRSF10 occurs during the M phase of the cell cycle [17] or under stress condition [18], when general inhibition of splicing occurs. In particular, it was demonstrated that, under normal conditions, phosphorylated SRSF10 is a sequence-specific splicing activator, which promotes U1 and U2 snRNP assembly on target pre-mRNAs endowed with SRSF10-dependent exonic splicing enhancer (ESE) sequences [16]. Conversely, under stressful cellular conditions, as during heat shock, SRSF10 is rapidly dephosphorylated by PP1, while other SR proteins are maintained in phosphorylated state by SR protein kinases (SRPKs) [19]. Interestingly, during the stress response all SR proteins are similarly dephosphorylated by PP1. However, they are rapidly rephosphorylated by SRPKs, while SRSF10, which is a poor substrate for SRPKs, remains dephosphorylated. Under this condition, SRSF10 can still interact with the U1 snRNP, but in this case the interaction impairs its ability to recognize the 5′ splice site, thus resulting in splicing inhibition [18].

**Figure 1:**
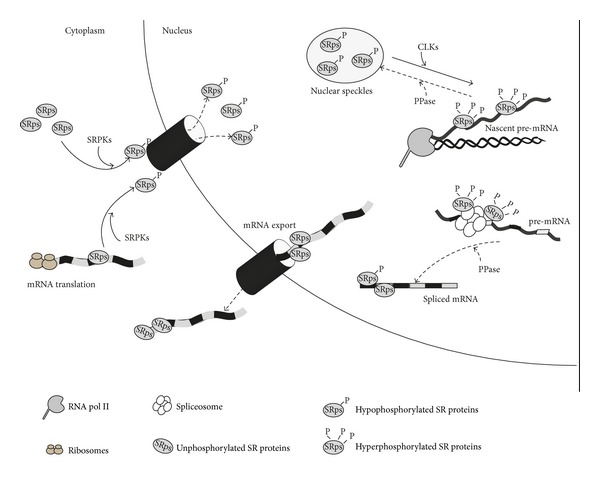
Phosphorylation-mediated regulation of SR proteins activity. Reversible phosphorylation of their RS domain profoundly affect SR protein (SRps) activity and subcellular localization. Newly synthesized SRps need SRPK-mediated phosphorylation in order to enter the nucleus and assemble in nuclear speckles. CLKs mediate SRps hyperphosphorylation and induce their release from nuclear speckles and their recruitment to transcription sites. Dephosphorylation of SRps is successively required for proper splicing catalysis. Moreover, dephosphorylated SRps facilitate export of spliced mRNA in the cytosol, where they enhance protein translation.

Phosphorylation of the RS domain can also dictate SR protein subcellular localization, by affecting both their intranuclear localization and their nucleocytoplasmic shuttling. In interphase cells, SR proteins are enriched in interchromatin granules called nuclear speckles, which are enriched in factors involved in pre-mRNA processing and RNA transport (reviewed in [20]). The recruitment of SR proteins to nascent pre-mRNAs from these sites of storage is regulated by their phosphorylation (Figure 1); indeed, it has been shown that phosphorylation of the RS domain is a prerequisite for their recruitment to transcription sites *in vivo *[21]. This modification plays also an important role in the regulation of nucleocytoplasmic shuttling of SR proteins. For instance, phosphorylation of SR proteins in the cytoplasm is required for their nuclear import [22]. On the other hand, dephosphorylation of the RS domain is essential for their translocation to the cytoplasm during mRNP maturation (Figure 1) [23]. Interestingly, dephosphorylation of SRSF1 and SRSF7 (previously known as 9G8) enhances their interaction with the export receptor TAP, thereby favoring also the export of their target mRNAs [24, 25]. Furthermore, SRSF1 translational activity is increased by dephosphorylation of its RS domain (Figure 1) [26]. Phosphorylation has therefore a great impact also on the splicing unrelated functions in which many SR proteins are involved (reviewed in [27]).

**Figure 2:**
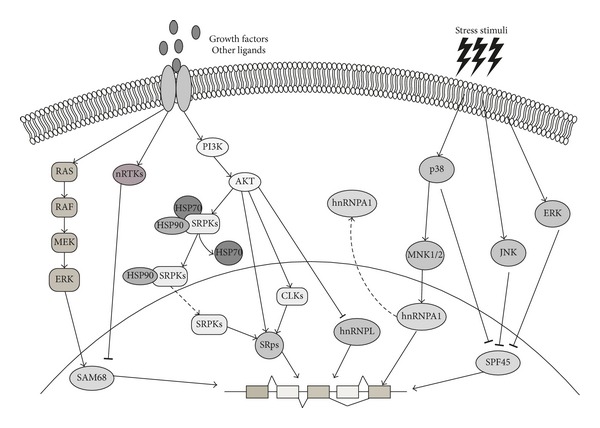
Signaling-activated kinases regulate splicing factor activity. Various extracellular cues, like growth factors or stress stimuli, activate different signal-transduction cascades impinging on protein kinases that in turn phosphorylate RBPs, thereby modulating their splicing activity. SAM68 splicing activity is inversely regulated by ERKs and nRTKs, which, respectively, activate and inhibit its splicing activity. The PI3 K-AKT pathway regulates the activity of several SR proteins both directly or by phosphorylating and modulating the activity and localization of CLKs and SRPKs. Stress signal-activated kinases, like JNK or p38, can both modulate splicing factor localization, like for hnRNPA1, or activity, like for SPF45 (see text for details).

Ser/Thr phosphorylation represents an important regulative process not only for SR proteins but also for hnRNPs and other splicing factors. For instance, hnRNP A1 is phosphorylated by the mitogen-activated protein kinase (MAPK) p38 in response to stress conditions (Figure 2), thus causing its cytoplasmic translocation and consequent modulation of hnRNP A1-sensitive AS events [28, 29]. Similarly, phosphorylation of the SR-like protein TRA2-*β* by CLK2 induces its relocalization into the cytoplasm, thus reducing its ability to bind its own mRNA and regulate its splicing [30]. For other splicing factors, instead, Ser/Thr phosphorylation affects the splicing activity by modulating their interaction with other proteins. For instance, phosphorylation of hnRNP L reduces its interaction with the U2AF65 subunit of the U2 auxiliary factor [31, 32]. The function of some splicing factors can be influenced also by Tyr phosphorylation. A well-documented example in this sense is SAM68, a member of the signal transduction and activation of RNA (STAR) family of RBPs (reviewed in [33]). Tyr phosphorylation by SRC-family kinases (SFKs) caused the accumulation of SAM68 in nuclear granules, named SAM68 nuclear bodies (SNBs) [34, 35]. Moreover, it was shown that this posttranslational modification negatively affected the interaction of SAM68 with hnRNP A1 and with the *BCL-X* pre-mRNA, thus impairing its ability to promote splicing of this target gene [35]. On the other hand, Ser/Thr phosphorylation of SAM68 by the MAPKs ERK1/2 was reported to increase splicing of the variable exons in the *CD44* gene [36, 37]. Notably, SAM68 represents an interesting example of how Ser/Thr and Tyr-phosphorylation may have opposite impact on the splicing activity of an RBP toward a target pre-mRNA (Figure 2). This was formally shown by studying its effect on the *CCND1* gene. Increased expression of SAM68 promotes splicing of the cyclin D1b variant of the *CCND1* gene in prostate cancer cells. This activity is further enhanced by activation of the RAS/ERK pathway and counteracted by SFKs [38]. In both cases, the effect was due to modulation of the affinity for RNA, as ERK-dependent phosphorylation increased binding of SAM68 to intron 4, whereas SFK-dependent phosphorylation abolished it (Figure 2) [38]. Thus, activation of signaling pathways can indirectly modulate AS events through posttranslational modification of selected splicing factors (see also later).

## 4. Splicing Factor Kinases

Phosphorylation of spliceosomal components and splicing factors is mediated by numerous protein kinases. Some of these kinases, such as the SRPK and CLK families, are specifically devoted to this function, whereas others also participate to signal transduction pathways or phosphorylate distinct primary substrates in addition to the splicing factors. Herein, we will review the kinases whose ability to influence splicing decisions has been better characterized. For convenience, we will classify them as SR-protein specific kinases; signaling-activated splicing kinases, and “atypical” splicing factor kinases. 

## 5. SR-Protein Specific Kinases

### 5.1. SR-Protein Kinases (SRPKs)

The first SR protein kinase identified was SRPK1, which was isolated from mitotic cells, and it was described to phosphorylate SR proteins and to promote their release from nuclear speckles during the G2/M phase of the cell cycle [39]. However, SRPK1 is present and active also in interphase cells. SRPK1 is the prototype of the SRPK family, which also includes the two homologous SRPK2 and SRPK3 proteins. SRPKs are characterized by a bipartite catalytic domain separated by a unique spacer sequence (reviewed in [40]) and are mainly localized in the cytoplasm of mammalian cells. This is due to the presence of a strong cytoplasmic retention signal localized in the spacer domain [41] and of their interaction with the molecular chaperones HSP70 and HSP90, which in complex seem to favor the folding of SRPKs into an active state [42]. However, SRPKs can translocate into the nucleus of cells under several conditions, such as during the G2/M phase of the cell cycle [39], or after osmotic stress [42], or as a consequence of activation of the epidermal growth factor (EGF) signal transduction pathway [43]. Due to this dual localization, SRPKs can phosphorylate SR proteins both in the nucleus and in the cytoplasm, thus affecting several aspects of their function. SRPK-mediated phosphorylation of SR proteins in the cytoplasm is necessary to ensure SR proteins nuclear import (Figure 1) [44], as it enhances their interaction with the specific transportin SR2 [22, 45]. SRPK nuclear activity promotes release of SR proteins in the nucleoplasm from the nuclear speckles [46]. For instance, several reports suggest that SRPK-mediated phosphorylation of SRSF1 is essential for its nuclear localization and the resulting splicing activity triggered by stimulation of specific signaling pathways (i.e., IGF-1 and EGF treatments) [43, 47]. However, under conditions that strongly increase nuclear localization of SRPKs, such as under cellular stress, they can also induce nuclear speckles enlargement [42, 48]. Indeed, Zhong and colleagues showed that osmotic stress induced by sorbitol treatment can lead to a massive nuclear translocation of SRPK1, which causes hyperphosphorylation of SR proteins and inhibits their splicing activity toward the reporter *E1A* minigene [42]. These studies indicated that SRPK-mediated phosphorylation of SR proteins can finely tune their splicing activity in response to external and internal cues. 

### 5.2. Cyclin-Dependent Like Kinases (CLKs)

The cyclin-dependent like kinases (CLK1-4) represent the other prototypical family of SR protein kinases. They are characterized by a C-terminal kinase domain, with dual specificity, and an N-terminal RS domain, which allows their interaction with the SR proteins. CLKs colocalize with SR proteins in nuclear speckles, and their overexpression leads to hyper-phosphorylation of SR proteins and induces speckles disassembly [49]. Several studies reported the ability of CLKs to influence splicing events by regulating the subnuclear localization of SR proteins (Figure 1). In particular, the release of SR proteins from nuclear speckles induced by CLKs overexpression has been reported to modulate splicing of the *E1A* reporter minigene [50] and of the exon 10 of the *TAU* gene [51], whose aberrant regulation has been implicated in several neurodegenerative diseases. Recently it has been shown that CLKs also modulate the activity of splicing factors not related to the SR-protein family, such as SPF45. CLK-mediated phosphorylation of SPF45 interferes with its proteasomal degradation and enhances exon 6 inclusion of *FAS* by promoting binding of this splicing factor to the *FAS* pre-mRNA [52]. The nuclear localization of CLKs is one of the major differences between them and SRPKs, which are instead mainly cytosolic. Because of their different localization, CLKs and SRPKs can cooperate in regulating SR proteins subcellular localization. Indeed, it has been shown that SRPK1 interacts with SRSF1 and phosphorylates the N-terminal part (RS1) of its RS domain, a posttranslational modification that is essential for its assembly into nuclear speckles, whereas CLKs phosphorylate the C-terminal part (RS2) of its RS domain, thereby causing release of SRSF1 from the speckles [53]. Moreover, SRPKs and CLKs have also distinct substrate specificity, as SRPKs preferentially phosphorylate Ser-Arg sites, while CLKs have a broader specificity and can phosphorylate also Ser-Lys or Ser-Pro sites [54]. Therefore, even if apparently redundant, the coordinated activity of SRPKs and CLKs is crucial for correct splicing regulation. This was well illustrated by Nowak and colleagues, whose work highlighted how SR-proteins phosphorylation induced by these two families of kinases may differently control a single splicing event [55]. The vascular endothelial growth factor A (*VEGFA*) gene, a key regulator of angiogenesis, produces several isoforms by alternative splice-site selection in the terminal exon 8: proximal splice-site selection results in proangiogenic *VEGFxxx* isoforms, whereas distal splice-site selection results in antiangiogenic isoforms *VEGFxxxb*. Different growth factors inversely influence these splicing events by inducing in both cases phosphorylation of SR proteins. However, IGF-1 and TNF-*α* induced production of *VEGFxxx* through activation of SRPKs, whereas TGF-*β*1 enhanced *VEGFxxxb* production by activating CLKs [55].

## 6. Signaling-Activated Splicing Factor Kinases

AS represents a crucial step in the regulation of gene expression in eukaryotic cells. Therefore, its regulation needs to be finely integrated in the complex network of regulative mechanisms that allows the cell to modulate gene expression in response to the different physiological and pathological stimuli that are received from both the internal and external environment. In support of this notion, activation of signal transduction pathways has been shown to modulate AS in a large number of situations. However, while in some cases the mechanism(s) has been described, in other cases the transacting factors mediating the response are unknown. Here we will review signaling-activated kinases that can modulate AS by directly phosphorylating splicing factors or their regulators, such as the SRPKs or CLKs. 

### 6.1. AKT

The Ser/Thr kinase AKT, also known as PKB, is the hinge molecule of the phosphoinositide-3-kinase-protein kinase (PI3 K) signaling pathway, which transduces the signal of several growth factors and cytokines. Through the phosphorylation of its many nuclear and cytosolic targets, AKT can regulate a multitude of cellular processes, such as cell metabolism, proliferation, and survival (reviewed in [56]). To exert its multiple functions, AKT regulates different steps of the gene expression network, from transcription, to AS and translation. Indeed, several reports have highlighted the ability of AKT to directly and indirectly modulate the function of many RBPs. AKT phosphorylates both hnRNPs and SR proteins, which contain within their RS domain multiple AKT phosphorylation consensus sequences: RXRXX(S/T) [57]. By modulating their phosphorylation status, AKT regulates both splicing and splicing independent functions of hnRNPs and SR proteins. For example, AS of a four-exon cassette in the *CASPASE-9* gene allows expression of a proapoptotic splice variant (exon inclusion, *CASPASE-9a*) or an antiapoptotic splice variant, (exon skipped, *CASPASE-9b*). AKT-dependent phosphorylation of hnRNP L increases its affinity for exon 3 and induces expression of the antiapoptotic variant. Indeed, AKT-phosphorylated hnRNP L competes with hnRNP U for the binding to the mRNA and impairs its ability to promote the pro-apoptotic *CASPASE-9a* [58] (Figure 2). This is a clear example of how AKT may promote cell survival by regulating a key AS event. On the other hand, phosphorylation of hnRNP A1 by AKT has no effects on splicing, but it modulates the translational activity of this RBP. Following phosphorylation, hnRNP A1 loses its ability to promote IRES dependent translation of the *CCND1* and the *c-MYC* mRNAs [59]. In the case of SR proteins, AKT was shown to modulate both splicing and translational activity through phosphorylation. Growth factor-induced phosphorylation of SRSF1 and SRSF7 by AKT enhanced their ability to promote the inclusion of the *EDA* exon in the fibronectin mRNA and translation of the spliced mRNA [60]. One of the most characterized AS events regulated by AKT is affecting *PKC*β** pre-mRNA after insulin stimulation. This hormone induces splicing of the *PKC*β* II* isoform, which enhances insulin-stimulated glucose transport better than the *PKC*β* I* variant, even if they differ only for two residues in their C-terminus [61]. Insulin stimulation induces PI3K-dependent activation of AKT, which phosphorylates SRSF5 [62, 63], thus promoting *PKC*β* II* splicing. Furthermore, AKT phosphorylates CLK1 and enhances its activity (Figure 2). In turn, CLK1 phosphorylates SRSF4 (previously named SRp75) and SRSF6 (previously named SRp55), thus contributing to the *PKC*β* II * splicing regulation [64]. Recently, it has been suggested that AKT and CLK may also regulate SRSF5 splicing activity by affecting its nuclear localization, which was impaired when a CLK mutant that cannot be phosphorylated by AKT is expressed [65]. Importantly, this concerted regulation of SRSF5-dependent *PKC*β* II* splicing by AKT and CLK was essential for adypogenetic differentiation, thus providing physiological relevance for this signaling route.

AKT was also shown to regulate the activity of SRPKs. A recent work documented that EGF signaling induces a massive reprogramming of AS that depends on AKT-induced nuclear translocation of SRPKs [43]. In fact, AKT binding to SRPKs induces their autophosphorylation and dissociation from the HSP70 chaperone, which normally holds SRPKs into the cytoplasm, thus favoring their nuclear translocation guided by HSP90 (Figure 2). Once in the nucleus, SRPKs can phosphorylate SR proteins and modulate the splicing pattern of several genes. Thus, given its ability to modulate the activity of both regulators (SRPKs and CLKs) and effectors (SR proteins and hnRNPs) of AS, AKT stands up as a crucial player in the modulation of splicing in response to external cues, and this activity might represent a primary function of AKT in the regulation of multiple cellular processes.

### 6.2. Mitogen-Activated Protein Kinases (MAPKs)

MAPKs are a family of Ser/Thr kinases that transduce external signals into the cell and regulate many different cellular processes, such as metabolism, proliferation, survival, differentiation, and motility (reviewed in [66]). The MAPK family includes the extracellular regulated kinases (ERK 1/2), the c-Jun amino terminal kinases (JNK 1–3), p38 (*α*, *β*, *γ*, and *δ*), and ERK5 family. The role of MAPKs in these cellular processes is mediated by regulation of protein activity and stability and by modulation of gene expression, which also occurs through AS. The first evidence of MAPK-modulated splicing came from studies on the regulation of AS of the CD44 gene, which encodes for the extracellular receptor for hyaluronic acid, a key component of the extracellular matrix. The *CD44* gene is characterized by a block of variable exons (v2–v10) embedded between ten constant exons; the inclusion of the variable exons into the mature transcript modulates CD44 protein interaction with its substrate, thus significantly affecting cell adhesion, migration, and proliferation (reviewed in [67]). The inclusion of the variable exon v5 in the mature mRNA of *CD44* upon T-cell activation is dependent by the RAS-RAF-MEK-ERK signaling cascade [68]. The target of this pathway is SAM68, whose ability to promote exon v5 inclusion is increased by ERKs-mediated phosphorylation [36]. SAM68 interacts with the splicing factor U2AF65, and this interaction seems to enhance the recognition of the 3′ splice-site. Phosphorylation by ERKs reduces the affinity of the SAM68/U2AF65 complex to the *CD44* pre-mRNA, probably favoring the subsequent recruitment of other spliceosomal components [69]. SAM68 is not the only RBP regulated by ERK1/2. Furthermore, other MAPKs, like p38 or JNKs, are known to phosphorylate and modulate the activity of splicing factors. For example, it has been recently demonstrated that phosphorylation of the splicing factor SPF45 can be mediated by all the three families of MAPKs in response to different stimuli (e.g., oxidative stress activates ERK1/2 and JNK mediated phosphorylation, whereas UV-light induces p38 and JNK activity) [70] (Figure 2). These kinases phosphorylate SPF45 on two residues, Thr 71 and Ser 222; these posttranslational modifications inhibit SPF45-dependent exon 6 inclusion in the *FAS* gene, thus leading to the production of a dominant negative isoform of this death receptor [70]. 

Modulation of hnRNP A1 activity by p38 is another well characterized regulative phosphorylation event operated by a MAPK. Environmental stresses, such as osmotic stress or UV irradiation, induce p38 activation and phosphorylation of hnRNP A1, leading to the relocalization of this nuclear RBP into the cytoplasm, where it concentrates into discrete phase-dense particles, called stress granules (SGs) [29] (Figure 2). The nuclear exclusion of hnRNP A1 is the result of its reduced interaction with the transportin Trn1, which under normal conditions mediates its nuclear translocation [71], and leads to consequent modulation of hnRNP A1-dependent splicing events, which were tested using the *E1A* minigene reporter [28]. HnRNP A1 phosphorylation is mediated by the p38 effectors MAP kinase signal-integrating kinases (MNK1/2) [28], which can also regulate the translational activity of this splicing factor. It was observed that the increase in TNF-*α* protein production following T-cell activation relies on MNK-mediated phosphorylation of hnRNP A1. However, in this cellular context, phosphorylation of hnRNP A1 does not affect its localization, but it rather lowers its affinity for the AU-rich element (ARE) in the 3′UTR of the *TNF-*α** mRNA, thus probably relieving a translation repressive control and allowing enhanced TNF-*α* production [72].

 Thus, MAPKs can regulate different steps of mRNA processing through phosphorylation of several splicing factors, integrating in this way this complex regulative step of gene expression with the response of the cell to external cues. 

### 6.3. Tyrosine Kinases

Protein tyrosine kinases (PTKs) catalyze the transfer of a phosphate group from ATP to a tyrosine residue of their target proteins. PTKs may be classified in two different classes: the transmembrane receptors tyrosine kinases (RTKs) and the nonreceptor tyrosine kinases (nRTKs). PTKs mediate the phosphorylation of several proteins in response to both internal and external cues, leading to the modification of their activity or affecting their interaction with other proteins. Transduction pathways triggered by PTK activation affect gene expression, also at the level of AS, even though only a small number of splicing factors have been shown to be regulated by Tyr-phosphorylation. Among these few RBPs, the members of the STAR proteins family, and in particular SAM68, stand out (Figure 2). In many STAR family members, the RNA binding domain is flanked by regulatory regions, like proline-rich or tyrosine-rich sequences, which mediate their interaction with the Src Homology 3 (SH3) and SH2 domains of other proteins, including PTKs. For instance, the breast tumor kinase (BRK) is a nRTKs that interacts in the nucleus with a proline rich region of SAM68 through its SH3 domain. BRK-dependent phosphorylation of SAM68 reduces its RNA binding affinity [73]. Analogously, BRK phosphorylates also the SAM68 homologous proteins SLM-1 and SLM-2, reducing their affinity to the RNA also in this case [74]. SAM68 is also substrate of FYN, another soluble nRTKs. In this case, it was also shown that Tyr-phosphorylation interfered with SAM68-dependent splicing of the *BCL-X* and *CCND1* genes [35, 38]. FYN-dependent phosphorylation reduced the affinity of SAM68 for these target RNAs and affected its interaction with different proteins, such as hnRNP A1, thereby affecting the outcome of AS events [35, 38]. Tyr-phosphorylation also influences the splicing activity of the nuclear RBP YT521-B, which can also be elicited by several nRTKs such as FYN, SRC, or c-ABL [75, 76]. This posttranslational modification induces translocation of YT521-B from the nuclear YT bodies, where it normally resides, to the nucleoplasm. Phosphorylated YT521-B shows reduced ability to modulate splice-site selection of different targets, in association with a reduced binding to their mRNA, possibly because the nucleoplasmic translocation distances YT521-B from the effective sites of pre-mRNA processing [76].

### 6.4. cAMP-Dependent Protein Kinase (PKA)

Increased intracellular levels of the second messenger cyclic adenosine 3′,5′-monophosphate (cAMP) lead to the activation of the cAMP-dependent protein kinase (PKA), which transduces the signals of many hormones, growth factors, and neurotransmitters [77]. PKA is a tetrameric protein, composed of two regulatory subunits (R) and two catalytic subunits (C): binding of cAMP to the R subunits leads to their dissociation from the C subunit and activation of the kinase [77]. Activated PKA phosphorylates several effectors, including transcription factors, ion channels, and metabolic enzymes, thus influencing multiple cellular functions. PKA activity is also regulated by interaction of the R subunits with the PKA-anchoring proteins (AKAPs); AKAPs maintain PKA in specific subcellular compartments and in proximity of its substrates, thus retaining PKA activity where it is needed. The first evidence of a possible involvement of PKA in the regulation of AS came from the observation that a fraction of the C subunit translocates into the nucleus, colocalizes with SRSF2 (previously reported as SC35) in splicing speckles, and phosphorylates several SR proteins, at least *in vitro* [78]. Localization of the C subunit in nuclear speckles seems to be related to its interaction with the C-subunit binding protein HA95 [78] and to the SR protein SRSF17A, which was shown to be a novel AKAP required to anchor PKA C subunit in splicing speckles [79]. Importantly, modulation of *E1A* reporter minigene splicing by SRSF17A is dependent on its interaction with PKA [79]. Moreover, nuclear PKA itself is able to modulate AS of the *E1A* reporter minigene, even in the absence of the cAMP stimulation [78]. 

Several stimuli that increase the cAMP intracellular levels were shown to affect AS events through phosphorylation of both SR proteins and hnRNPs by PKA. For example, it was demonstrated that forskolin, which stimulates the synthesis of cAMP, modulates AS of exon 10 of the *TAU* gene [80, 81]. Notably, activated PKA affects the activity of two SR proteins, SRSF1 and SRSF7, which inversely modulate exon 10 splicing: SRSF1 promotes exon 10 inclusion, whereas SRSF7 prevents it. However, PKA-dependent phosphorylation of SRSF1 enhances its activity [80] whereas it inhibits SRSF7 [81], thus globally favoring exon 10 inclusion. 

PKA is also able to modulate AS of genes that are crucial for neuronal differentiation, through the phosphorylation of hnRNP K. After phosphorylation by PKA, hnRNP K shows higher binding activity to its target mRNAs with respect to its competitor U2AF65; this mechanism impairs the recognition of the 3′ splice site and leads to the skipping of its target exons [82]. On a broader view, hnRNP K target motifs are found in many genes involved in neuronal differentiation and in neurological diseases [82]. These pieces of evidence suggest that PKA mediated regulation of hnRNPs and SR proteins activity may be an important player in the complex network of regulative mechanisms that finely control AS events during neuronal development (reviewed in [83]). Although cAMP and PKA are usually involved in cell differentiation, their contribution to cancer has also been demonstrated. It will be interesting to investigate whether PKA-dependent modulation of AS also occurs in genes with relevance to human cancer.

## 7. Other Kinases

In this section, we will describe the regulative activity of some proteins that showed an unexpected kinase activity towards splicing factors, so that they cannot be included in any of the classes described previously. Some of these kinases were known to have other specific substrates different from splicing factors, for others, instead, the kinase activity was totally unpredicted. 

### 7.1. DNA Topoisomerase I

The first of these atypical kinases to be described was the DNA topoisomerase I, whose best known function is to relieve both positive and negative DNA supercoils ensuring correct DNA topology during transcription, DNA replication and repair (reviewed in [84]). Despite the absence of a canonical ATP binding site, DNA topoisomerase I was shown to phosphorylate SR proteins, in particular the prototypic SRSF1, within their RS domain [85]. This phosphorylation event can significantly affect SR proteins modulatory activity on AS events. Indeed, it has been demonstrated that cells deficient for this enzyme show a general status of hypophosphorylation for the SR proteins, which correlates with an impaired regulation of several AS events, whereas constitutive splicing results unaffected [86]. Moreover, treatment with a selective inhibitor of the kinase activity of DNA topoisomerase results in reduced phosphorylation levels for SR proteins, which in turn leads to a defective spliceosome assembly and alterations in the splicing pattern of several genes [87]. As it is now well established that pre-mRNA splicing occurs cotranscriptionally, it has been suggested that this double activity of DNA topoisomerase I could be one of the mechanisms ensuring the correct coordination between DNA transcription and splicing [88]. Indeed, DNA topoisomerase I activity is fundamental to solve DNA supercoils generated by RNA pol II progression along the DNA template and might simultaneously ensure a regulated splicing factor activity through their phosphorylation.

### 7.2. Dual-Specificity Tyrosine-(Y)-Phosphorylation Regulated Kinase 1A (DIRK1A)

Another protein kinase able to modulate the splicing activity of SR proteins is DIRK1A (dual-specificity tyrosine-(Y)-phosphorylation regulated kinase 1A). This dual-specificity protein kinase autophosphorylates on Tyr, Ser, and Thr residues but phosphorylates substrates only on Ser or Thr residues (reviewed in [89]). The human DYRK1A gene maps to chromosome 21, and it is ubiquitously expressed in adult and fetal tissues, with high levels of expression in the brain. DYRK1A is supposed to play a major role during neuronal development, through its interaction with several cytoskeletal, synaptic, and nuclear proteins (reviewed in [89]). Several SR proteins were shown to interact with DYRK1A. Indeed, DYRK1A has been reported to colocalize with SRSF2 in nuclear speckles, and its overexpression induces the disassembly of this subnuclear structures [90]. Alteration of subcellular localization of the SR proteins phosphorylated by DYRK1A seems to be the main mechanism by which this kinase regulates the splicing activity of its target factors. For instance, phosphorylation of SRSF1 and SRSF7 by DYRK1A induces their cytoplasmic translocation [91, 92], whereas phosphorylation of SRSF2 and SRSF6 causes their dissociation from nuclear speckles [93, 94]. For each of these splicing factors the mislocalization induced by DYRK1A impaired their ability to modulate the inclusion of exon 10 of the *TAU* gene, thus shifting the splicing balance toward the exclusion of this exon. 

### 7.3. Fas-Activated Serine/Threonine Kinase (FAST)

Fas-activated serine/threonine kinase (FAST) is a constitutively phosphorylated Ser/Thr kinase, which undergoes rapid dephosphorylation after the binding of Fas ligand to its receptor Fas, an interaction that triggers T-cell apoptosis. It was known that dephosphorylated FAST was able to interact with and phosphorylate the RBP TIA1 [95], but the functional relevance of this interaction in the regulation of the splicing process remained unknown for a long time. It was later discovered that phosphorylation of TIA1 by FAST regulates its ability to promote the inclusion of exon 6 of the *FAS* gene [96]. Phosphorylated TIA1 enhances U1 snRNP recruitment to *FAS* pre-mRNA, thus favoring the recognition of this variable exon. Inclusion of exon 6 into the *FAS* mRNA favors the production of a proapoptotic isoform of this gene, suggesting that FAST and TIA1 take part to a positive regulative circuitry that enhances Fas-dependent apoptosis once activated. Furthermore FAST is also endowed with an intrinsic splicing activity, independent from TIA1 [97]. It was observed that FAST can modulate the splicing of the *FGFR2* reporter gene in the same direction of TIA1, favoring the inclusion of exon III b but independently from this RBP. Thus, FAST can directly and indirectly affect splicing, and it would be interesting to determine how many targets are influenced by this kinase in T cells. 

### 7.4. Aurora Kinase A (AURKA)

AURKA was identified in a high-throughput siRNA screening for factors involved in the regulation of AS of two apoptotic genes: *BCL-X* and *MCL1* [98]. Among several regulators identified by the screen, authors noticed a peculiar enrichment for proteins involved in the regulation of the cell cycle. They focused their study on AURKA, a kinase involved in the regulation of centrosomal splitting that is frequently upregulated in cancers, where it is supposed to promote aneuploidy [99]. AURKA was demonstrated to positively regulate splicing of the antiapoptotic variant *BCL*-*X*_*L*_ through stabilization of SRSF1. Cells depleted of AURKA showed reduced levels of SRSF1, which then resulted in increased levels of the *BCL*-*X*_*S*_ pro-apoptotic variant. Moreover, since AURKA is activated at the G2/M phase of the cell cycle, the authors suggested that this kinase links *BCL-X* splicing regulation to cell cycle progression. These observations suggest that, in addition to the effects on centrosome duplication, upregulation of AURKA can favor neoplastic transformation also by promoting antiapoptotic splice variants. 

## 8. Splicing Factor Kinases in Cancer and Other Human Diseases

Due to the important role played by the AS process in the control of gene expression, any alterations of its regulation can profoundly modify important cellular processes, thus resulting in a potential cause of disease (reviewed in [100]). Altered expression, activity, or subcellular localization of splicing factor kinases can be among the causes of the aberrant splicing events associated to several diseases, particularly neurodegenerative pathologies and cancer.

Aberrant inclusion of exon 10 of the *TAU *gene is a well-known example of pathogenetic splicing event, caused by the deregulated activity or expression of splicing factor kinases. TAU protein is a microtubule associated protein, which controls assembly and stability of microtubules. Exon 10 of the *TAU* gene encodes for one of the four microtubule binding domain repeats (R) of the TAU protein and regulates its affinity for microtubules and, consequently, its ability to induce their polymerization. Alternative inclusion of exon 10 leads to the production of either 4R-tau (inclusion) or 3R-tau (exclusion), and equal levels of these two isoforms seem to be essential for normal function of the human brain. Alteration of the normal ratio 1 : 1 between the 4R and the 3R isoform, in both directions, has been observed in several cases of Alzheimer's disease (AD); moreover, nearly half of the mutations in the *TAU *gene associated with FTDP-17 (frontotemporal dementia with parkinsonism linked to chromosome 17) affects exon 10 splicing, both inhibiting or promoting its inclusion, strongly suggesting that a proper regulation of this splicing event is essential for the maintenance of the healthy balance between 4R and 3R isoforms (reviewed in [101]). Several reports have highlighted or suggested a strong correlation between aberrant splicing of the exon 10 of the *TAU *gene in tauopathies and deregulated activity of the kinases regulating this splicing event. Stamm's group, for example, observed an increased production of an inactive isoform of the CLK2 kinase in the brain of AD patients, which correlated with increased inclusion of *TAU* exon 10. This observation suggested that the CLK2-dependent phosphorylation of SR proteins and the SR-like protein TRA2-*β* is required for the correct regulation of this splicing event [102]. Another kinase supposed to be involved in the altered regulation of *TAU *splicing is PKA, which also promotes the inclusion of the exon 10 of this gene through the phosphorylation of different SR proteins [80]. As reduced levels of PKA-C*α* have been observed in AD brains [103], it has been speculated that the lack of its activity may participate in the alteration of the normal balance between the 3R and 4R splice variants of *TAU* [81]. As described in previous section, the kinase DYRK1A exerts an important regulation on the AS of the *TAU *gene, thus strongly suggesting that its increased dosage due to the trisomy of chromosome 21 could be the main cause of the early onset of tauopathies in patients with Down syndrome [103]. Modulation of *TAU *gene splicing is a very attractive potential therapeutic target for treatment of tauopathies (reviewed in [104]); since protein kinases regulate this splicing event and are involved in tauopathy pathogenesis, targeting the activity of these kinases should be certainly considered in the development of future approaches for the treatment of these pathologies.

Upregulation and/or misregulated activity of splicing kinases are often associated to cancer development. This has been widely reported for SRPK1, which is overexpressed in several cancer types, such as pancreatic carcinomas [105], breast and colon carcinomas [106], and lung cancer [107]. Moreover, increased SRPK1 levels positively correlate with tumor grade [106] and are associated with higher resistance to chemotherapeutic treatments [105, 108]. Through modulation of selective splicing events, SRPK1 may allow cancer cells to enhance their proliferative, invasive, and angiogenetic potential. For example, in pancreatic, breast, and colon cancer cells SRPK1 promotes the generation of specific splice variants of the *MAP2K2* gene, which sustained higher activity of the MAPK pathway [106]. Recently, SRSF1 mediated splicing of the *MNK2b* isoform of the *MKNK2* gene has been correlated with resistance to gemcitabine treatment in pancreatic cancer cells [109]; since SRPK1 is upregulated in this cancer type and promotes cell survival, it would be interesting to evaluate whether this kinase contributes to the SRSF1-induced prosurvival pathway. A similar regulation has been described in Wilms Tumor, wherein SRPK1 promotes the production of the proangiogenic isoform *VEGF165* of the *VEGFA* gene through the phosphorylation and nuclear translocation of SRSF1 [48]. In these nephroblastomas tumors SRPK1 transcriptional upregulation is driven by the mutated transcription factor WT1, and its splicing activity is fundamental for the high levels of vascularization required by these tumors [48]. Importantly, the physiological relevance of SRPK1 for angiogenesis has been demonstrated, as injection of an SRPK1/2 inhibitor reduced it in a mouse model of retinal neovascularization, suggesting that targeting AS through their upstream regulator could be a potential tool to target pathological angiogenesis in cancer [48].

 Several signal transduction kinases, whose activity is often deregulated by neoplastic transformation, exert their oncogenic activity in part through the aberrant regulation of splicing events. For instance the MAPK pathway, which is frequently hyperactivated in tumors, can promote the acquisition of an invasive and migratory phenotype by modulating the AS pattern of the cell adhesion molecule *CD44*. In fact, it has been shown that hepatocyte growth factor (HGF) can induce cell migration of cancer cells by promoting this splicing event, as a consequence of induced ERK1/2-mediated phosphorylation of SAM68, induced by the MET receptor signaling pathway [110]. Also epithelial-to-mesenchymal transition (EMT), which is crucial for the invasiveness of cancer cells, is regulated by AS events that are sensitive to activation of the MAPK pathway. Indeed, production of the constitutively active Δ*RON *splice variant of the *RON* oncogene, the extracellular receptor for HGF, leads to EMT in colorectal cancer cells [111]. This splicing event is promoted by the upregulation of SRSF1. Remarkably, under conditions that favor EMT, epithelial cells release soluble factors that activate the ERK1/2 pathway. This in turn causes phosphorylation and activation of SAM68, which causes retention of a cryptic intron in the 3′UTR of the SRSF1 mRNA, reducing the amount of the nonsense-mediated-decay (NMD) targeted splice variant and enhancing expression of SRSF1 [112]. Thus, activation of the ERK1/2 pathway triggers a cascade of splicing events that culminate in a cellular response favoring cancer cell invasion. 

Activation of the AKT pathway has also been suggested to promote cancer cell survival through the regulation of specific splicing events. For example, it has been observed that hyperactivation of AKT through the RAS signaling pathway is implicated in the production of pro-survival splice variants of the *KLF-6* and *CASPASE-9 *genes in nonsmall-cell lung cancer and hepatocellular carcinoma, respectively, [113, 114]. In both cases, AKT induces SRSF1 phosphorylation, enhancing its ability to promote *KLF-6SV1* and *CASPASE-9b *isoforms. These observations strongly suggest a primary role for this splicing regulatory activity in the oncogenic potential of AKT. 

## 9. Protein Phosphatase Regulating Splicing Factors

In the previous paragraphs, we have broadly described the importance of a proper balance between phosphorylation and dephosphorylation events in the regulation of the pre-mRNA splicing process. Therefore, even if this review focuses primarily on the activity of the numerous kinases involved in this regulation, a brief description of the protein phosphatases counteracting their activity is also required for a comprehensive overview of the phosphorylative regulation of splicing.

PP1 and PP2A were the first Ser/Thr phosphatases whose activity was demonstrated to be necessary for splicing catalysis [5, 6]. PP1 and PP2A are required for the later steps of the splicing reaction, in particular for the second transesterification reaction, whose accomplishment is favored by dephosphorylation of U5 (U5-156 kDa) and U2 (SAP155) snRNP components [9]. In particular, PP1-mediated dephosphorylation of SAP155 is favored by the nuclear inhibitor of PP1 (NIPP1). NIPP1 is a nuclear regulatory subunit of PP1, enriched in nuclear speckles [115], known to interact with several splicing regulators, like CDC5L or the same SAP155 [116, 117]. In particular, NIPP1 stimulates PP1-mediated dephosphorylation of SAP155 by facilitating the interaction between the phosphatase and its substrate [10]. In subsequent studies, PP2C*γ* was also demonstrated to be important for the splicing process, as it was shown to be physically associated with the spliceosome, and its enzymatic activity was necessary for the early steps of spliceosome assembly [118]. 

Ser/Thr phosphatases are important regulators of both constitutive and AS events, as it was suggested by pioneering studies showing alternative 5′ splice selection after addition of PP1 in splicing assay *in vitro *[119]. Furthermore, PP2C*γ* was shown to interact with the RBP YB-1 and to modulate AS of the *CD44* gene [120], while PP1 was demonstrated to interact with a short motif RVXF motif within the RRM of several splicing factors, like SRSF1, SRSF9 (previously known as SRp30C), and the SR-like protein TRA2-*β* [121]. Dephosphorylation of TRA2-*β* by PP1 positively modulates its dimerization and its interaction with partner proteins, like SRSF1. Moreover, PP1 regulates alternative splice selection in TRA2-*β* target mRNAs like the *SMN2 *gene [121]. Exclusion of the exon 7 of *SMN2 *gene, combined with the primary deletion of *SMN1* gene, is the cause of the spinal muscular atrophy (SMA) [122]. TRA2-*β* promotes the inclusion of the exon 7 of *SMN2* favoring the production of a functional full length protein. TRA2-*β* splicing activity is enhanced by inhibition of PP1 activity [121] and, surprisingly, by activation of PP2A [123]. Indeed, the Stamm's group found that a class of compounds derivative from cantharidin (a well-known phosphatase inhibitors) activates PP2A, which in turn dephosphorylates TRA2-*β* on Thr33, favoring inclusion of exon 7 [123]. These observations suggest the possibility to develop new protein phosphatase inhibitors that could be used for the therapeutic correction of the splicing defects occurring in neurodegenerative diseases like SMA.

Modulating protein phosphatases' activity in order to manipulate pathogenetic splicing events has been suggested as a potential therapeutic tool also for cancer treatment. Indeed, it has been shown that genotoxic agents inducing apoptosis in cancer cells act through the generation of ceramide and activation of PP1, which in turn promotes the formation of the proapoptotic *BCL*-*X*_*S*_ and *CASPASE-9b *splice variants [124]. On the other hand, it has been shown that the proapoptotic activity of synthetic ceramides, like C6 pyridinium ceramide, is instead associated with activation of PP1 and the consequent reduced phosphorylation of several splicing factors and modulation of several splicing events [125]. These observations underline the importance of the regulated activity of protein phosphatases for proper regulation of the splicing process and strongly suggest the possibility to develop new molecules targeting their activity, which could be used for the therapeutic correction of the splicing defects occurring in several human diseases.

## 10. Concluding Remarks

Increasing evidence points out to a key role of misregulation of AS in the cellular transformation process. Cancer-specific splice variants can potentially be used as accurate diagnostic and prognostic markers, as it was recently highlighted by genome-wide studies [126, 127]. Targeting the splicing process represents, therefore, an attractive therapeutic target for cancer treatment, and it is currently under intense investigation. Therapeutic modulation of AS is mainly realized through RNA-based technologies (reviewed in [128]) or through chemical reagents inhibiting spliceosome activity (reviewed in [129]). The RNA-based technologies exploit antisense oligonucleotide masking specific sequence elements to splicing factors and/or the spliceosome [130], whereas chemical approaches make use of drugs that directly target the activity of spliceosomal components, as for examples spliceostatin A, which inhibits the SF3b subunit of the U2 snRNP, thereby modulating the AS of genes important for cell cycle control [131].

Considering the important control exerted by protein kinases on AS, modulation of their activity represents a potential approach for the development of new drugs targeting RNA splicing in cancer therapy. These suggestions are supported by recent reports highlighting the high efficacy of SRPK1/2 inhibitors in reducing angiogenesis through the negative modulation of the AS of the proangiogenic splice variant *VEGFxxx* gene [48]. Considering the great impact that SRPKs have on the splicing activity of SR proteins and the large number of AS events that they regulate, modulation of SRPK activity could be a powerful tool in the emerging field of splicing-modulating therapies. It is also important to mention that SRSF1, a well-known target of SRPKs, is upregulated in human cancers and functions as an oncogene [132]. For the same reasons, CLKs are a fascinating chemotherapeutic target too, and important efforts are being made for the realization of selective and efficient CLKs inhibitors [133]. 

Signal-transduction pathways able to modulate the phosphorylation status of SR proteins or the activity of other RBPs represent another potential druggable target for RNA splicing modulation. For example, it has been recently shown that amiloride, a well-known diuretic, can reduce proliferative and invasive properties of both hepatocellular carcinoma and leukemia cancer cells by inducing hypophosphorylation of SR-proteins [134, 135]. Genome-wide exon array analysis has demonstrated that amiloride treatment induces the modulation of a large number of AS events, and, in particular, it negatively regulates protumoral splice variants of several genes, such as the antiapoptotic *BCL*-*X*_*L*_ or proinvasive Δ*RON*. Reduced phosphorylation levels of AKT and ERKs were observed after amiloride treatment, suggesting that this drug reduces SR protein phosphorylation through inactivation of these kinases [135]. 

Deregulation of signal-transduction pathways in cancer cells is a general feature, and much effort has been made in order to develop chemotherapeutic agents that efficiently inhibit the activity of the kinases mediating the intracellular transduction of these signals, such as AKT or kinases of the MAPK and SRC families (reviewed in [136–138]). As many of these inhibitors are already in clinical practice, and many of them are undergoing promising clinical trials, it would be very interesting to understand whether their antiproliferative and cytotoxic effects could be partly due to their ability to interfere with AS events regulated by these kinases. Even more attractive is the possibility to exploit protein kinase inhibitors to selectively affect splicing decisions in order to restore in cancer cells a normal, nonpathological AS pattern. 

Shedding light on the expression, structure, and functions of the kinases regulating the activity of splicing factors is therefore an important step for a comprehensive understanding of the molecular mechanisms regulating pre-mRNA processing, which is essential for the rational design of future therapies targeting the aberrant AS process in cancer and other human diseases.
